# Novel assays to investigate the mechanisms of latent infection with HIV-2

**DOI:** 10.1371/journal.pone.0267402

**Published:** 2022-04-27

**Authors:** Michael D. Lu, Sushama Telwatte, Nitasha Kumar, Fernanda Ferreira, Holly Anne Martin, Gayatri Nikhila Kadiyala, Adam Wedrychowski, Sara Moron-Lopez, Tsui-Hua Chen, Erin A. Goecker, Robert W. Coombs, Chuanyi M. Lu, Joseph K. Wong, Athe Tsibris, Steven A. Yukl

**Affiliations:** 1 Department of Medicine, University of California, San Francisco (UCSF), San Francisco, CA, United States of America; 2 Department of Medicine, San Francisco VA Health Care System, San Francisco, CA, United States of America; 3 Brigham and Women’s Hospital, Harvard Medical School, Boston, MA, United States of America; 4 Department of Laboratory Medicine and Pathology, University of Washington, Seattle, WA, United States of America; George Mason University, UNITED STATES

## Abstract

Although there have been great advancements in the field of HIV treatment and prevention, there is no cure. There are two types of HIV: HIV-1 and HIV-2. In addition to genetic differences between the two types of HIV, HIV-2 infection causes a slower disease progression, and the rate of new HIV-2 infections has dramatically decreased since 2003. Like HIV-1, HIV-2 is capable of establishing latent infection in CD4^+^ T cells, thereby allowing the virus to evade viral cytopathic effects and detection by the immune system. The mechanisms underlying HIV latency are not fully understood, rendering this a significant barrier to development of a cure. Using RT-ddPCR, we previously demonstrated that latent infection with HIV-1 may be due to blocks to HIV transcriptional elongation, distal transcription/polyadenylation, and multiple splicing. In this study, we describe the development of seven highly-specific RT-ddPCR assays for HIV-2 that can be applied to the study of HIV-2 infections and latency. We designed and validated seven assays targeting different HIV-2 RNA regions along the genome that can be used to measure the degree of progression through different blocks to HIV-2 transcription and splicing. Given that HIV-2 is vastly understudied relative to HIV-1 and that it can be considered a model of a less virulent infection, application of these assays to studies of HIV-2 latency may inform new therapies for HIV-2, HIV-1, and other retroviruses.

## Introduction

HIV-2 is endemic to West Africa [1, 2]. While the incidence of HIV-2 infection is dramatically lower than that of HIV-1, there are up to 2 million people worldwide living with HIV-2 [3, 4]. It is important to distinguish between HIV-1 and HIV-2 infections because the disease progression and management differ. HIV-2 is characterized by a longer asymptomatic stage [5], a slower decline of CD4^+^ T cells, lower mortality related to AIDS (Acquired Immunodeficiency Syndrome), and lower person-to-person transmission rates than HIV-1 [6–8]. Individuals living with HIV-2 generally have a slower progression to AIDS, which is mediated by faster recognition and activation of the immune system in response to HIV-2 infection [9]. Globally, HIV-1 has had a steady number of new infections since 1988, while the number of new HIV-2 infections has declined in the same period, leading to growth and spread of HIV-1 but depletion of HIV-2 [9]. This decrease has been linked to lower HIV-2 transmissibility, lower viral fitness, lower capacity of HIV-2 Vif to counteract antiviral APOBEC3G/F editing [10] and more HIV awareness [11]. Effective treatment options are also available for HIV-2, but HIV-2 exhibits intrinsic resistance to non-nucleoside reverse transcriptase inhibitors (NNRTIs) and the fusion inhibitor T-20 (enfuvirtide) [12]. In addition, reduced susceptibility to NRTIs, protease inhibitors, and entry inhibitors has been reported in some HIV-2 isolates [13–17]. Despite these differing features, end-stage disease (AIDS) from both HIV-1 and HIV-2 follows a similar clinical course [18, 19].

Although the reasons why HIV-2 causes slower disease progression are not fully understood, a few contributing factors have been identified in the limited previous research. First, HIV-2 uses a wider variety of receptors, including C-C chemokine receptor type 1 (CCR1), CCR2, CCR3, C-X-C motif Chemokine Receptor 6 (CXCR6), and G-protein coupled receptor 15 (GPR15, or Brother of Bonzo [BOB]). Broader receptor usage allows infection of a wider range of T-cells and antigen-presenting cells [20, 21], which can lead to earlier detection of the virus as well as faster and more robust immune responses. HIV-2 capsids also bind more strongly than HIV-1 capsids to the non-POU domain-containing octamer-binding protein (NONO), leading to DNA sensing through the cGAS pathway and stimulation of a strong interferon-gamma (IFN-y) response [22, 23]. HIV-2 also has an additional accessory protein, VPX, which targets and degrades SAMHD1, a protein responsible for blocking the replication of HIV-1 in dendritic cells. VPX allows HIV-2 to replicate in dendritic cells, providing another pathway for earlier immune system detection [24, 25]. Lastly, contrary to HIV-1 infection, CD4^+^ and CD8^+^ T-cells maintain their poly-functionality during HIV-2 infection [26, 27]. Collectively, these mechanisms lead to an earlier detection of HIV-2 infection, allowing for a more robust immune response, better control of viral replication, and less CD4^+^ T-cell depletion.

HIV-2 infection is characterized by lower viremia than HIV-1, but studies are conflicting as to the burden of proviral DNA. Some studies report that HIV DNA levels are similar for HIV-1 and HIV-2 infection [28–31], while others demonstrate smaller reservoirs associated with HIV-2 infection [32], particularly in individuals infected with HIV-2 group B [33]. Studies in untreated individuals with HIV-2 suggest that despite generally lower plasma viral loads, levels of cell-associated gag mRNA are similar to those observed in HIV-1 infection, indicating significant HIV-2 transcription [34]. In contrast, mutations in the HIV-2 LTR [33] and lower levels of HIV-2 tat mRNA relative to HIV-1 infected individuals suggest transcriptional differences between HIV-1 and HIV-2 that may contribute to the lower pathogenicity of HIV-2 [34]. Given the slower progression to AIDS and lower mortality with HIV-2 infection [5], comparison of HIV-1 and HIV-2 persistence and expression may lead to new hypotheses about treatment and eradication of both viruses, especially since HIV-2 is vastly understudied.

The major barrier to curing HIV-1 and HIV-2 is the ability of these viruses to establish a latent infection in some CD4^+^ T-cells and possibly other cells. These latently infected cells harbor an integrated provirus and do not constitutively produce virions, but can be induced by activation to produce infectious virions [35–38]. Intensive study of HIV-1 has implicated numerous factors associated with viral persistence, including proviral amplification through clonal expansion [39–44], accumulation of proviral defects [45–47], and the influence of integration into actively-transcribed genes [48] or non-genic chromosomal regions [49]. Blocks at various stages of HIV expression are likely important molecular mechanisms underlying drivers of latency. In HIV-1 infection, these blocks have been attributed to transcriptional interference [48, 50], proviral integration into heterochromatin [51], epigenetic modification [52–57], absence of host transcription initiation factors [48, 58] or elongation factors [59–62], nucleosome positioning [55, 61, 63, 64], insufficient Tat activity [65–71], antisense transcription [72] and post-transcriptional processes, such as insufficient Rev levels [73], nuclear export defects [74, 75], RNA interference [76–79], or blocks to splicing [80, 81]. Although less is known about HIV-2 latency or expression, one study comparing the 5’ untranslated regions (5’UTR) for HIV-1 and HIV-2 found that the intrinsic structural motifs present in the HIV-2 trans-activation of transcription (TAR) region slow translation from HIV-2 genomic RNA, resulting in lower Gag production during viral replication [82].

To investigate the degree to which blocks at different stages of HIV-1 transcription may contribute to latency in ART-treated individuals, we previously developed a panel of seven reverse transcription droplet digital PCR (RT-ddPCR) assays targeting different regions of HIV-1 RNA along the genome [83]. Droplet digital PCR was selected for its ability to provide “absolute” quantification, its lower susceptibility to the effects of sequence mismatches or inhibitors, and the fact that it is more precise than quantitative PCR (qPCR) at low copy numbers [84, 85]. Using these RT-ddPCR assays, we determined that blocks to HIV-1 transcriptional elongation, completion, and splicing are the major reversible mechanisms that inhibit HIV-1 expression in blood cells from ART-treated individuals, while HIV-infected cells in the gut and cervix are also characterized by a stronger block to HIV transcriptional initiation.

Reasoning that the mechanisms of latency may or may not be the same for HIV-2, we employed a similar strategy to design primer/probe sets targeting different HIV-2 RNA regions that indicate transcriptional interference (read through transcripts) or progression through stages of HIV-2 transcriptional initiation, elongation, completion, and splicing. Application of these assays to clinical samples will advance our understanding of the mechanisms of HIV-2 latency and may assist in efforts towards new therapies aimed at disrupting latent infection with HIV-2, HIV-1, and other retroviruses.

## Materials and methods

### Primer design and selection

**Fig 1:**
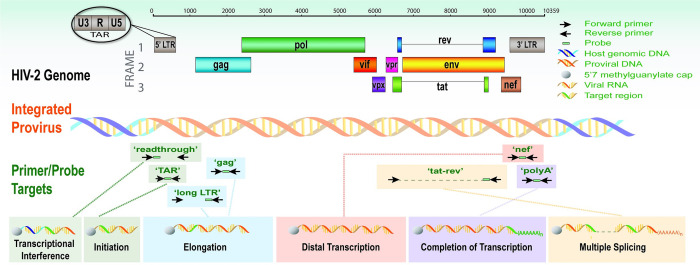
Schematic representation of the HIV-2 genome and targets for HIV-2 transcription profiling assays. The schematic depicts the genetic organization of the HIV-2 genome, proviral DNA, and the HIV-2 ‘transcription profiling’ primer/probe sets that target specific RNA sequence regions: ‘read-through’, ‘TAR’, ‘Long LTR’, gag’, ‘nef’, ‘polyA’ and ‘tat-rev’. The transcriptional feature targeted is listed below the respective assay. Adapted from Telwatte et al., PLoS Pathogens 2018 and HIV sequence compendium 2010.

To measure the degree to which blocks at different stages of transcription may contribute to HIV-2 latency, we designed a panel of 7 RT-ddPCR assays to measure “read-through” (U3-U5), initiated (TAR), 5’ elongated (R-U5-pre-gag; “Long LTR”), mid-elongated and unspliced (gag), distally transcribed (nef), completed/polyadenylated (U3-polyA; “polyA”), and multiply-spliced (tat-rev) HIV-2 transcripts (Fig 1) [83]. For a given region of interest (e.g.: U3-U5 for read-through transcripts), the Los Alamos National Lab HIV Sequence Database was used to design primers and probes to target the areas that were most conserved and to match the consensus of all available sequences from individuals infected with HIV-2 group A or group B [86] (Table 1). In cases where group A had a consensus that differed sufficiently from group B and too many degenerate bases would be required to match both, primer/probe sequences were chosen to match the consensus of group A. Two primer/probe sets were designed for Long LTR, polyA, and gag RT-ddPCR assays; preliminary experiments were used to select the best performing set for subsequent testing.

**Table 1:** Primer/probes for HIV-2 assays.

Assay	Name	Sequence (5’–3’)	MAC239 Position^1^
Read-through	RT/PolyA_F^2^	CCTCATACTTTCTGTATAAATGTACCCGCT	474–503
	RT/PolyA_P^3^	TTCAGTCGCTCTGCGSAGAGGCT	516–538
	Readthru_R^4^	GCGGCGACTAGGAGAGATGG	710-729RC^5^
TAR	TAR_F	GAGGTTCTCTCCAGCACTAGCAG	556–578
	TAR_P	AGAGCCTGGGTGTTC	581–595
	TAR_R	AGCACTGGTGAGAGTCTAGCA	598-618RC
Long LTR	LongLTR_F	CTTGCTTAAAGACCTCTTAATAAAGCTGCC	652–682
	LongLTR_P	CCAGGCGGCGACTAGGAGAGATGGGA	708-733RC
	LongLTR_R	GGGCGCCAAYCTGCTAGGGA	810-829RC
Gag	Gag_F	GCGCGAGAARCTCCGTCTTG	1057–1076
	Gag_P	TAGGTTACGGCCCGGCGGAAAGA	1109–1131
	Gag_R	AATTCATTCGCTGCCCACACAAYATGTT	1147-1174RC
Nef	Nef_F	ACACCAAGAGTACCACTAAGARCAATGAC	9383–9411
	Nef_P	ATTGGCAGTAGAYATGTCACATTT	9418–9441
	Nef_R	TCACTGTAAAACATCCCTTCCAGTCC	9458-9483RC
PolyA	RT/PolyA_F	CCTCATACTTTCTGTATAAATGTACCCGCT	474–503
	RT/PolyA_P	TTCAGTCGCTCTGCGSAGAGGCT	516–538
	PolyA_R	TTTTTTTTTTTTTTTTTTTTTTTTTTTGCTTCTAA	PolyA+ 3’ end of R RC
Tat-Rev	Tat-Rev_F	AAGGGGCTCGGGATATGKTATG	6509–6530
	Tat-Rev_P	CCCGGTTATVTCCAACAGATCCMTATCC	8788–8815
	Tat-Rev_R	CTTCTGTTTCTTCKYTGGCTGGCTGT	8829-8854RC

All probes feature a FAM fluorophore and MGB quencher.

1: MAC239 was chosen because this is the reference used by the QuickAlign program from the Los Alamos HIV Sequence Database.

2: F = Forward primer.

3: P = Probe.

4: R = reverse primer.

5: RC = reverse complement.

### Preparation of supernatant viral RNA (ROD) standard

To measure the performance of the new HIV-2 assays that detect genomic (unspliced) viral RNA, an HIV-2 genomic RNA standard was prepared from the supernatant of cells infected with ROD, an infectious HIV-2 laboratory virus derived from a clinical isolate [1, 87]. The ROD virus was passaged in peripheral blood mononuclear cells (PBMC) and harvested at day 7 post infection. Supernatant virus was clarified using two centrifugation steps (500 *xg*, 30 minutes). Virion-associated RNA from the supernatant was isolated and prepared as previously described [88]. Briefly, ROD supernatant (400 μL diluted in PBS) was treated with DNAse I and RNAse A to eliminate free nucleic acids and subjected to three freeze/thaw cycles to lyse any residual cells but not virions [89, 90]. RNA extraction was performed using the QIAgen Viral RNA Mini Kit as per manufacturer’s guidelines, with 100 μL elution volume per column. The number of HIV-2 copies in the extracted RNA was quantified in replicate using the Abbott m2000sp/rt platform adapted for quantification of HIV-2 RNA in plasma. This method, which was developed at the University of Washington and validated for clinical use, targets a region of the LTR conserved in group A and B HIV-2 [91–93]. The supernatant viral RNA standard was subsequently diluted in nuclease free water, reverse transcribed, and used to evaluate the performance of the new ddPCR assays for HIV-2. The “expected copies” in each ddPCR well were calculated using the value from the established Abbott qPCR assay, dilution factor, volume added to the reverse transcription (RT) reaction, and proportion of the RT added to each ddPCR well.

### Preparation of synthetic, in vitro transcribed (IVT) viral RNA standard

The HIV-2 supernatant viral RNA standard would be expected to contain mostly unspliced genomic HIV-2 RNA, which should not be detected by the assays for read-through or multiply-spliced HIV-2 RNA. Therefore, we designed a 2886bp synthetic HIV-2 viral RNA standard that begins with the 5’ U3 region, splices from D1 to exon 1 of tat and between exons 1 and 2 of tat and rev, and encodes a polyA tail at the end of the 3’ R region. This standard contains the regions targeted by the read-through, TAR, Long LTR, nef, polyA, and multiply-spliced tat-rev assays (but not gag, which is found only in unspliced HIV RNA). The HIV-2 sequence was chosen to match the consensus of HIV-2 group A using the Los Alamos National Lab HIV Sequence Database and was constructed between the Nhe I and Xba I cloning sites of the plasmid pcDNA3.1(+). This plasmid was used as an HIV-2 DNA standard and also used for *in vitro* transcription of an HIV-2 RNA standard.

To prepare the latter, the plasmid was linearized by digestion with Xba I, and the HIV-2 sequence was *in vitro* transcribed using the T7 RiboMAX™ Express Large Scale RNA Production System (Promega, Madison, WI). After *in vitro* transcription, the RNA was purified by the QIAgen RNeasy kit with on-column DNAse. The concentration of the extracted RNA (synthetic IVT standard) was measured by ultraviolet (UV) spectrophotometry (NanoDrop ND-1000 instrument, Thermo Fisher) and the molecular weight was used to calculate the expected number of molecules per μL. The length, integrity, and concentration of the IVT standard was also confirmed using the Agilent Bioanalyzer RNA 6000 Nano assay (Agilent, Santa Clara, CA). In addition, the copies of HIV-2 RNA were independently quantified using the Abbott m2000sp/rt platform adapted for the clinical quantification of HIV-2 [92]. The IVT standard was subsequently diluted in nuclease free water and used for RT and ddPCR. The “expected copies” in each ddPCR well were calculated using the value from the Nanodrop, dilution factor, volume added to the reverse transcription (RT) reaction, and proportion of the RT added to each ddPCR well.

### Reverse transcription

To mitigate bias towards reverse transcription of the 3’ end (as expected with poly-dT alone), the 5’ end (as expected with random hexamers), or any one region (as expected with specific reverse primers), reverse transcription (RT) reactions were primed using both random hexamers and poly-dT. RT reactions were performed in a 50 μL mixture containing 5 μL of 10× SuperScript III buffer (Invitrogen), 5 μL of 50 mM MgCl_2_, 2.5 μL of random hexamers (50 ng/μL; Invitrogen), 2.5 μL of 50 μM poly-dT15, 2.5 μL of 10 mM deoxynucleoside triphosphates (dNTPs), 1.25 μL of RNAseOUT (40 U/μL; Invitrogen), 2.5 μL of SuperScript III RT (200 U/μL; Invitrogen), and water or one of the HIV-2 standards +/- cellular RNA from uninfected donor cells. Reactions were performed in a standard thermocycler with the following cycling conditions: 25°C for 10 min, 50°C for 50 min, and an inactivation step at 85°C for 5 min. Aliquots of cDNA from the RT reaction were then added to ddPCR reactions containing primers and probe specific for a given HIV-2 RNA region.

### Droplet digital PCR

Primer/probe sets were tested using the QX100 (Bio-Rad, Hercules, CA) droplet digital PCR (ddPCR) system. Each HIV-2 assay (primer/probe set) was tested in at least duplicate using aliquots from the same common RT reaction. The total reaction volume (20 μL) consisted of 10 μL of ddPCR probe supermix (no deoxyuridine triphosphate; Bio-Rad, CA, USA), 900 nM of primers (Table 1; Life Technologies, CA, USA), 250 nM of probe (Life Technologies; Table 1), and 5 μL of cDNA. After droplet formation, the cDNA was amplified using a Mastercycler® Nexus (Eppendorf, Hamburg, Germany) with the following cycling conditions: 95°C for 10 min, 45 cycles of 30 s at 95°C and 59°C for 1 min, and a final droplet cure step at 98°C for 10 min (S1 Table). Droplets were read and analyzed using the QX100 Droplet Reader and QuantaSoft software (Bio-Rad, CA, USA) in the absolute quantification mode.

### Determination of HIV-2 assay sensitivity using plasmid DNA and *in vitro* transcribed HIV-2 RNA standards

Plasmid DNA encoding the read-through, multiply-spliced synthetic HIV-2 region was quantified by UV spectrophotometry (NanoDrop) and the molecular weight was used to calculate the number of molecules per μL. The plasmid HIV-2 DNA was added to ddPCR wells at expected inputs of 1−10^4^ copies/well in duplicate (10^4^, 10^3^ and 10^2^ copies) or quadruplicate (10 and 1 copy) prior to ddPCR.

Known concentrations of HIV-2 RNA were added to RT reactions and the resultant cDNA was added at expected inputs (per final ddPCR well) of 1−10^3^ copies/well in duplicate (10^3^, 10^2^ and 10^1^ copies) or quadruplicate (1 copy) prior to ddPCR.

### Nucleic acid isolation from donor PBMC for “background” RNA

RNA and DNA were extracted from cryopreserved donor PBMC using TRIreagent according to the manufacturer’s protocol (Molecular Research Center), except that DNA was extracted using the back extraction buffer (4M guanidine thiocyanate, 50 mM sodium citrate, and 1 M Tris [free base]). RNA was resuspended in 25 μL of nuclease free water, measured on the NanoDrop instrument, and stored at -80°C until use. DNA was resuspended in 25 μL of QIAgen EB buffer (Qiagen, Hilden, Germany), incubated at room temperature overnight, and stored at -80°C until use.

### Assay sensitivity in the presence of background RNA

Additional experiments were performed using the standards and isolated donor PBMC RNA to determine each assay’s performance in the presence of “background” cellular RNA. Standards were diluted to the 10,000; 1,000; 100; 10; and 1 copies/5 μL before being added to 50 μL to 70 μL RT reactions with or without cellular RNA from PBMCs. RT products were then added to 20 μL ddPCR reactions and run as described above for each assay.

## Results

### Efficiency, sensitivity, and linearity of HIV-2 RT-ddPCR assays determined using HIV-2 plasmid DNA standard

**Fig 2:**
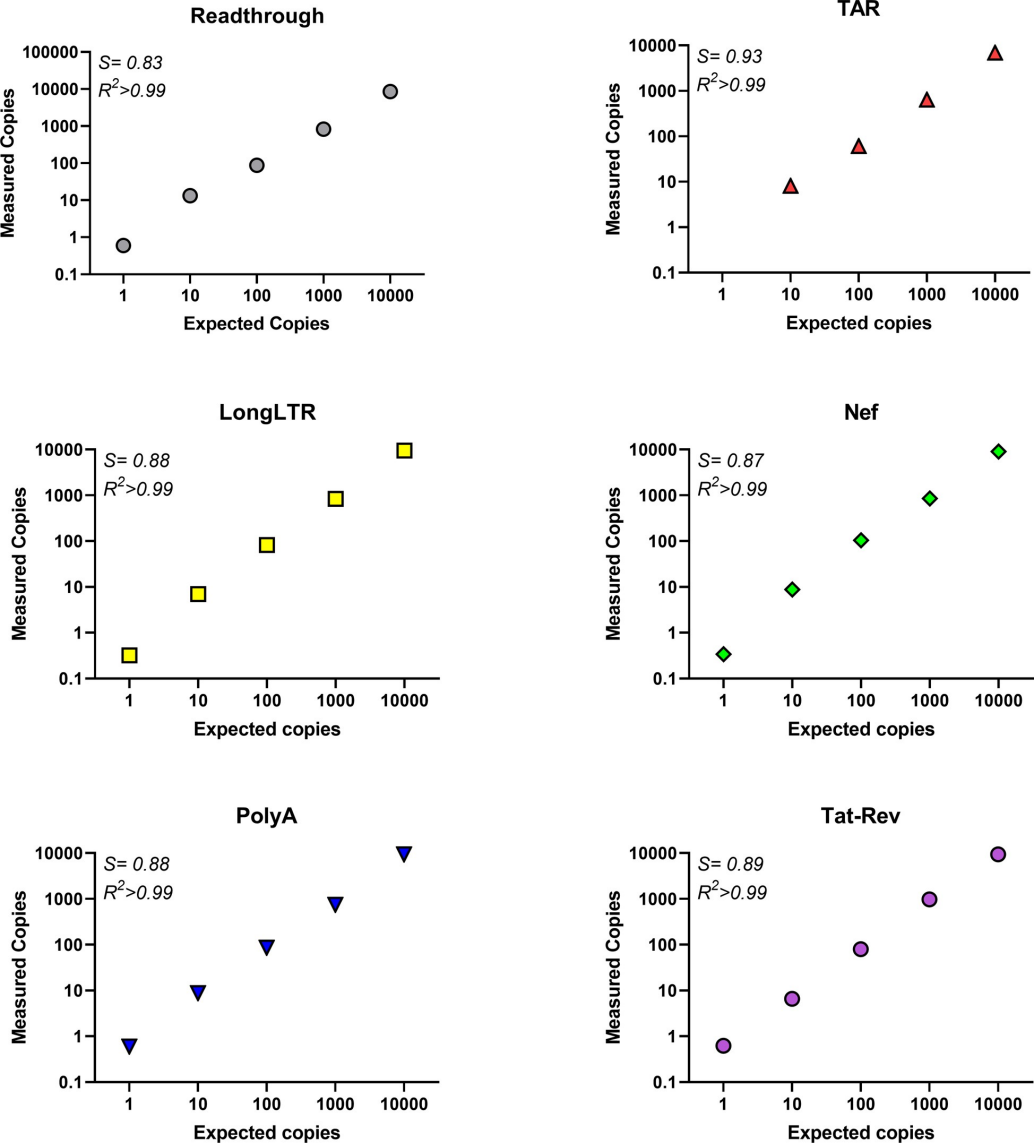
Efficiency and linearity of HIV-2 RT-ddPCR assays determined using HIV-2 plasmid DNA. Plasmid DNA was quantified by UV spectrophotometry (NanoDrop) and diluted (expected copies) to determine the absolute number of copies detected by each primer/probe set using ddPCR reactions (measured copies). Data represent average of duplicate/quadruplicate wells from a representative experiment. *S* = slope, indicating assay efficiency. Each primer/probe set was tested in at least two independent experiments.

The efficiency, linearity, and sensitivity of the new HIV-2 primer/probe sets for all regions except gag were measured using terminal dilutions of the synthetic HIV-2 plasmid (11,330 copies down to 0.11 expected copies). Each primer/probe set was consistently able to detect fewer than 10 copies, and as few as 1 copy in quadruplicate measurements (Fig 2). Assay efficiencies (slopes of measured vs. expected copies) were greater than ≥83% and similar for all assays (Readthrough = 0.83, TAR = 0.85, Long LTR = 0.88, Nef = 0.87, PolyA = 0.89, Tat-Rev = 0.88; Fig 2). All assays showed linear quantification (R^2^≥0.999) with a dynamic range of ≥4 log10. The gag coding region is absent from this construct and as such, the gag primer/probe set was not evaluated using plasmid DNA.

### Efficiency, sensitivity, and linearity of HIV-2 RT-ddPCR assays using synthetic IVT HIV-2 RNA standard

**Fig 3:**
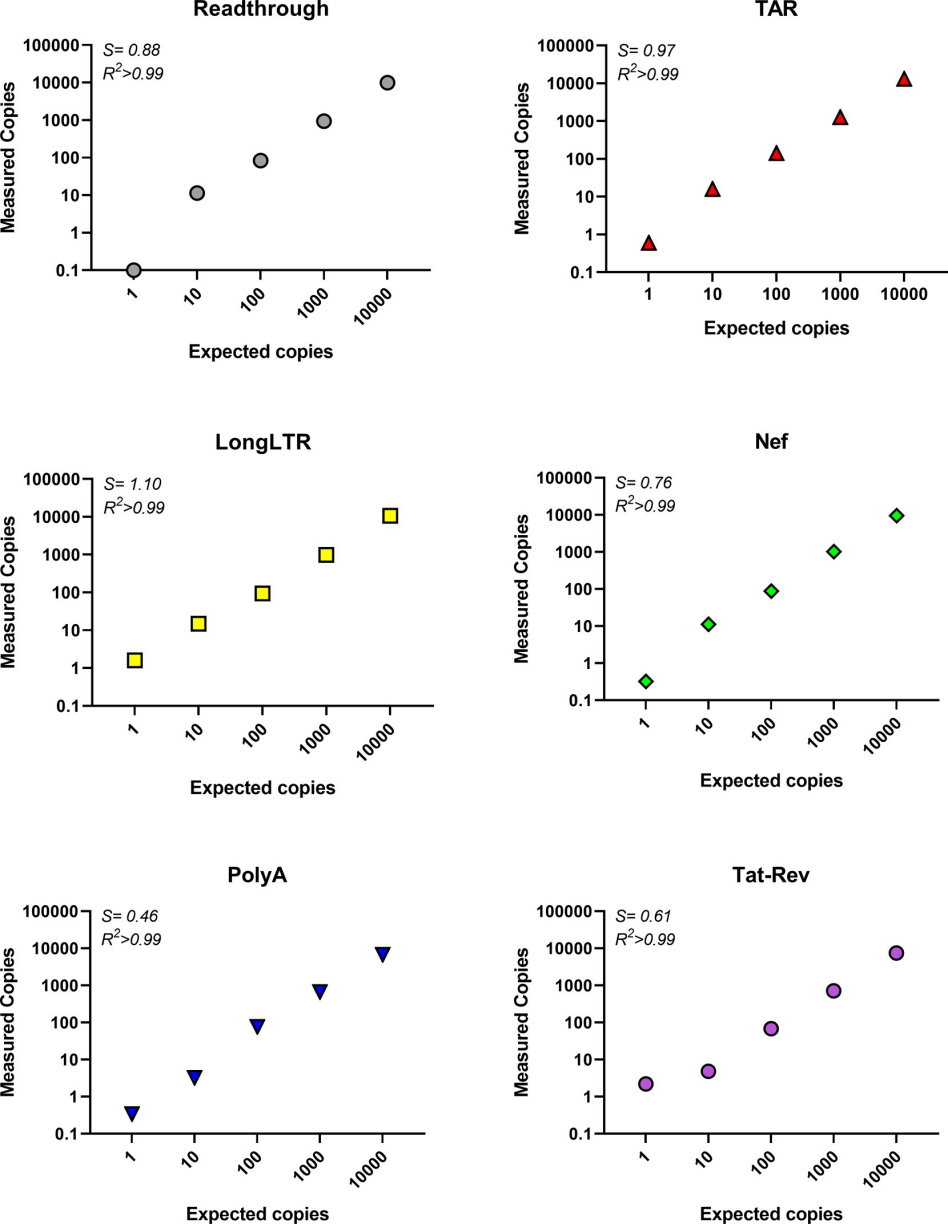
Efficiency and linearity of HIV-2 RT-ddPCR assays determined using *in vitro* transcribed (IVT) RNA. An RNA standard was prepared by *in vitro* transcription (IVT) from a HIV-2 plasmid and quantified by independent means (UV spectroscopy, the Agilent Bioanalyzer, and a clinical assay using the Abbott m2000sp/rt platform). Various inputs of the IVT RNA standard (which were used to calculate ‘Expected Copies’ per ddPCR well) were reverse transcribed. Replicate aliquots of cDNA were used to measure the absolute number of copies detected by each primer/probe set (‘Measured Copies’). Each primer/probe set was tested using expected inputs of 1−10^4^ copies per ddPCR well. Data represent average of duplicate wells from a representative experiment. *S* = slope, indicating assay efficiency for n = 2 independent experiments, R^2^ = coefficient of determination/goodness of fit of linear regression for n = 2 independent experiments.

All primer/probe sets except those for gag were also tested using standards prepared from *in vitro* transcribed (IVT) RNA generated from the custom-designed HIV-2 plasmid. Expected copy numbers were calculated using the RNA concentration and molecular weight. RNA was added to RT reactions to achieve final input copy numbers of 10^3^, 10^2^, 10^1^, 10^0^ and 10^−1^ copies/ddPCR well. All primer/probe sets could detect as few as 10 copies, while read-through, polyA and tat-rev were also detected in wells containing 1 copy (Fig 3). Assay efficiencies (slopes of measured vs. expected copies) were high but showed some variation between target regions (read-through = 0.85, TAR = 0.95, Long LTR = 1.14, nef = 0.76, polyA = 0.43, and tat-rev = 0.57; median = 0.77). All assays showed linear quantification (R^2^≥0.999) with a dynamic range of ≥4 log10. These validation experiments demonstrated that our selected primer/probe sets exhibit high efficiency and linearity using RNA that underwent reverse transcription and ddPCR.

### Performance of HIV-2 RT-ddPCR assays using viral supernatant RNA (ROD) standard

**Fig 4:**
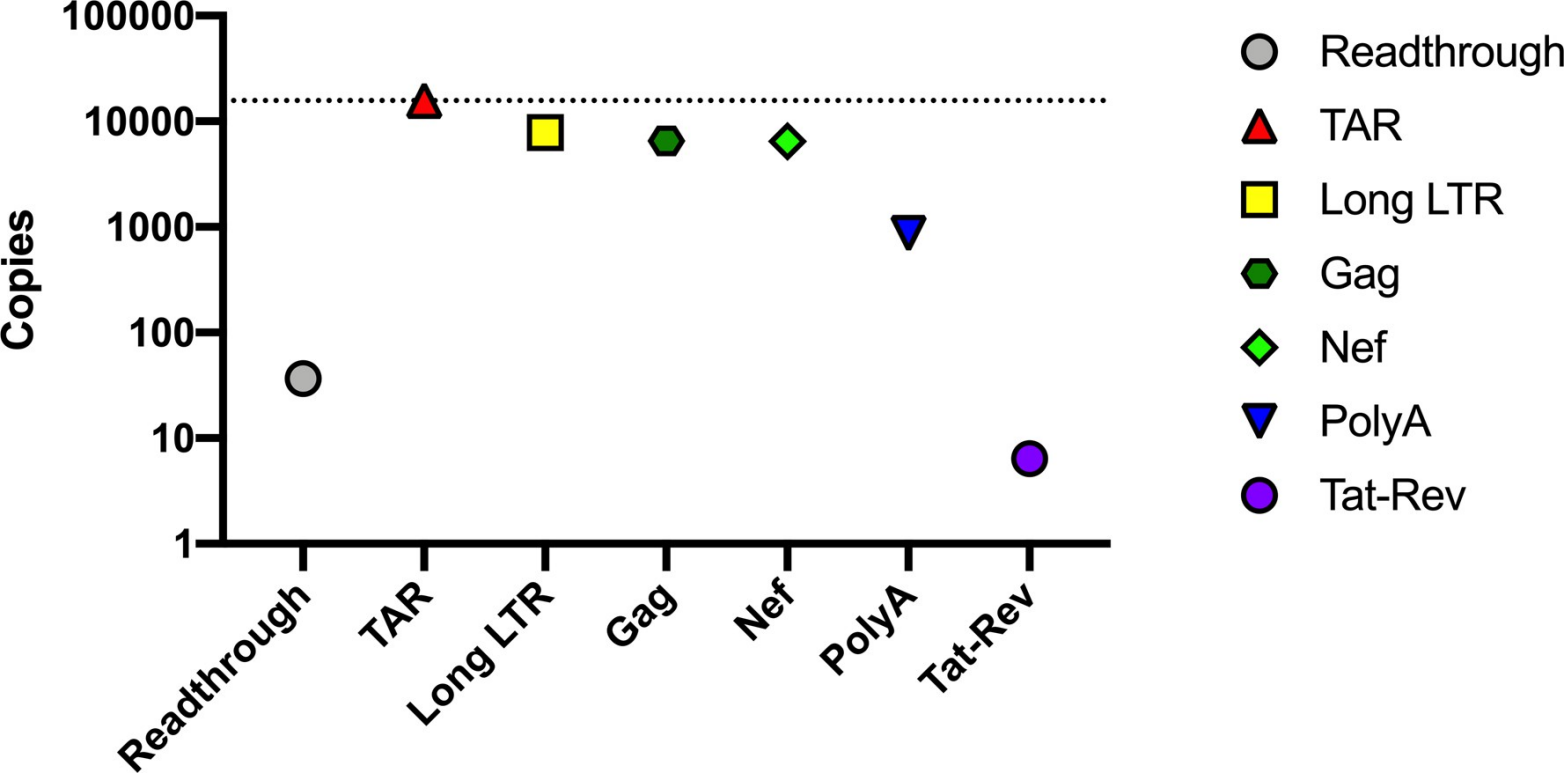
Levels of HIV-2 targets in viral supernatant RNA. The viral supernatant HIV-2 standard was quantified by a clinical assay [92] and a known input was added to a common RT reaction. cDNA was synthesized in this common RT reaction and subsequently divided equally between ddPCR wells for all HIV-2 targets to assess the efficiency of each primer/probe set.

The viral supernatant RNA (ROD) standard was used to further evaluate the performance of our new RT-ddPCR assays for HIV-2 TAR, Long LTR, gag, nef, and polyA RNA. Known inputs of the viral supernatant RNA standard (based on quantification by clinical assay) were added to a common RT reaction. The subsequent cDNA was divided among different assays, and each was measured in duplicate with an expected input of 15,714 copies/ddPCR well. Our TAR assay detected 15,800 copies, which is similar to the expected value from the clinical assay targeting the LTR region (Fig 4). The measured copies of Long LTR (7,840), gag (6,540), and nef (6,480) were approximately half that of the TAR region, consistent with the fact that every full-length genomic HIV-2 RNA contains 2 TAR regions. PolyA was detected at lower levels (868 copies), but it should be noted that the reverse primer of the polyA assay was designed to match the consensus from HIV-2 infected individuals and has multiple sequence mismatches with the ROD virus. Read-through and tat-rev transcripts were detected at even lower levels (37 and 6 copies, respectively), likely because these transcripts are not efficiently packaged into virions and most free viral RNA should be degraded by the freeze-thaw nuclease treatment used to prepare the standard.

### Primer/probe specificity for HIV-2 vs. HIV-1 using DNA standards

**Fig 5:**
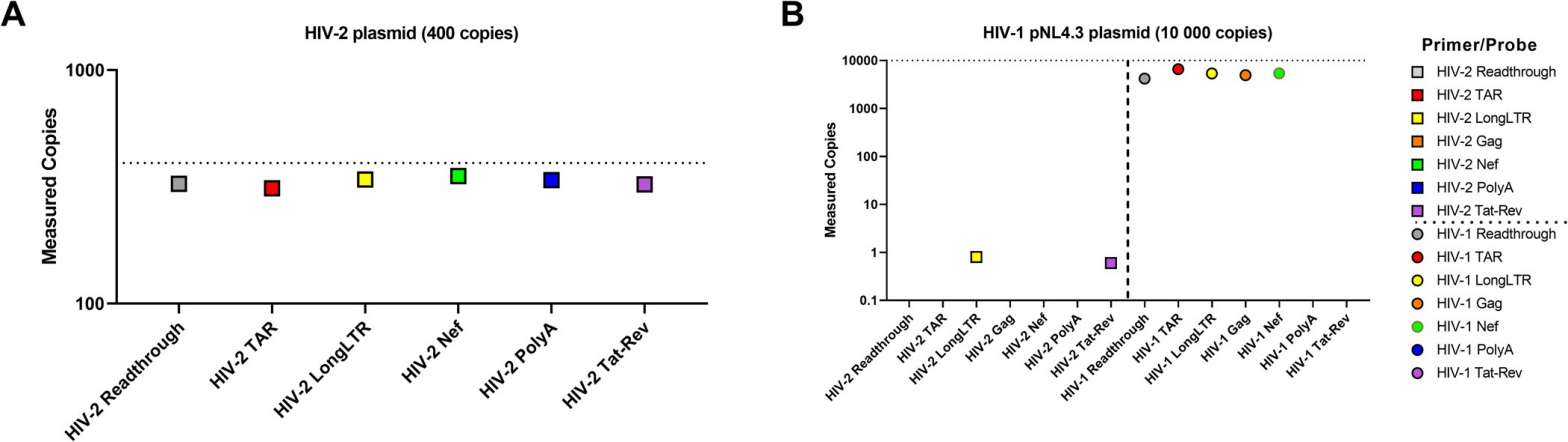
Specificity of HIV-2 primer/probes for HIV-2 DNA. (A) HIV-2 plasmid DNA was added at a constant input of 400 copies into duplicate reactions to measure each target (read-through, TAR, Long LTR, nef, polyA, and tat-rev). (B) HIV-1 plasmid (pNL4.3) was added at a constant input of 10,000 copies into each reaction to measure detection of both HIV-2 and HIV-1 targets.

Despite sharing genetic features with HIV-1, HIV-2 exhibits considerable sequence differences. Accordingly, it was hypothesized that our primer/probe sets would be highly specific for HIV-2 templates only. We assessed the specificity of each primer/probe set using inputs of either 400 copies of HIV-2 plasmid or 10,000 copies of HIV-1 (pNL4.3) plasmid DNA. At a 400-copy input of HIV-2 plasmid, all our HIV-2 primer/probe sets performed efficiently, detecting a median of 83% of the expected input (78%-88%; Fig 5A). As predicted, even at a high input of 10,000 copies of HIV-1 DNA, our HIV-2 primer/probe sets showed minimal false positives (no copies for read-through, TAR, gag, nef, or polyA, and only 1 copy for Long LTR and tat-rev; Fig 5B). In contrast, our HIV-1 primer/probes sets detected around 10,000 copies, in line with previously measured efficiencies [83].

### Assay efficiency is not adversely affected by background RNA

To determine whether our primer/probe sets are inhibited by the presence of cellular RNA (“background”), we performed an RT-ddPCR experiment using a constant input of 1,000 copies of HIV-2 IVT RNA standard in the presence or absence of 1 μg RNA from peripheral blood mononuclear cells (PBMC). In line with previous observations using other primer/probe sets [94], we found that the presence of background RNA was not inhibitory (S1 Fig). Instead, the detection of all HIV-2 targets actually improved in the presence of background RNA, consistent with our previous observations with SARS-CoV-2 targeting primer/probe sets [95]. No false positives were detected in wells containing donor PBMC RNA without HIV-2 RNA.

### Primer/probe specificity and false positive rates

To determine the non-specific reactivity of oligonucleotides (false positive rate) of our assays, each primer/probe set was tested using ‘no template’ controls (NTC). These reactions were performed with molecular-grade H_2_O, HIV-1 RNA (from pNL4-3), and RNA isolated from HIV-negative donor PBMC (S2 and S3 Tables). Identifying any HIV-1 non-specificity is important for the purposes of our assays because epidemiology and clinical management differ for HIV-1 and HIV-2, and since HIV-2 can be found in dual-infection with HIV-1, albeit at lower incidences [96, 97]. Except for Long LTR and tat-rev, both of which only had 1 false positive droplet amidst tens of thousands of analyzed droplets, no other HIV-1-related false positives were observed. For the H_2_O and PBMC NTCs, false positive detections were only seen in Long LTR (2 of 14 wells) and TAR (1 of 15 wells), all of which were from donor PBMCs (S2, S3 Tables). Again, these results were amidst tens and even hundreds of thousands of analyzed droplets.

### Assay reproducibility

S4 Table shows the coefficient of variation (% CV) between experiments (inter-experiment) and within experiments (intra-experiment) for each assay at inputs of 100 and 1,000 copies of the HIV DNA plasmid standard or the IVT HIV RNA standard.

## Discussion

We present a novel panel of primer/probe sets targeting key regions of the HIV-2 transcriptome that may provide insight into the transcriptional blocks which govern HIV-2 latency. We selected droplet digital PCR rather than conventional qRT-PCR because ddPCR allows for absolute quantification (without the need for external calibration), is more forgiving of sequence mismatches in primer/probe sequences, is likely more precise at lower copy numbers, and provides similar sensitivity and reproducibility [83, 85]. We selected 5′-6-carboxyfluorescein dye (FAM) and a 3′-Minor Groove Binder (MGB) group for our probes given MGB forms highly-stable duplexes with single-stranded DNA, permitting the use of shorter probes and yielding higher specificity [98]. Our validation studies demonstrated that all primer/probe sets could detect as few as 1–10 copies using plasmid DNA carrying the cognate sequence and demonstrated linearity over 4 orders of magnitude (R2>0.999 for all; Figs 2 and 3). Using plasmid DNA, we found that PCR efficiencies were high and very similar across all targets (range = 83–89%; Fig 2).

Using synthetic RNA, we found that read-through, TAR, Long LTR and nef primer/probe sets performed with similar efficiencies to those observed using plasmid DNA, indicating that reverse transcription (RT) does not considerably diminish the ability to detect input copies. We did observe lower efficiency in the polyA and tat-rev primer/probe sets using the IVT standard compared to HIV-2 plasmid DNA (polyA: 43% vs. 89% and Tat-Rev: 57% vs. 88%, respectively), suggesting that the RT reaction could contribute some loss in detection capability. However, in the presence of background RNA (up to 1μg PBMC RNA), which is a closer approximation of clinical samples, we found that this loss of sensitivity of polyA and tat-rev was reduced (S1 Fig).

Each of our HIV-2 assays demonstrated high level sensitivity (down to 1–10 copy inputs) over >4 orders of magnitude (see Figs 2 and 3) and inter-assay reproducibility (S4 Table), with minimal false positives using non-template controls (NTCs) (see S2 and S3 Tables). For studies involving people with HIV (PWH), it is imperative to distinguish HIV-2 from HIV-1. We found that our assays are highly-specific for HIV-2 (Fig 5). At 10,000 copies of HIV-1 (NL4-3), the HIV-2 read-through, TAR, gag, nef, and polyA assays exhibited no detection, while Long LTR and tat-rev had only 1 positive droplet across duplicate wells. With inputs of H_2_O or donor PBMC RNA, only Long LTR and TAR had any false positives (Long LTR: 2/14 wells, and TAR: 1/15 wells; S3 Table). Together, these data suggest that our assays are highly-specific and sensitive for HIV-2, with minimal false positives. These defined characteristics are extremely important for studying clinical samples because HIV-2 infected individuals may have low levels of HIV-2 RNA.

Our primer/probe sets performed better in the presence of additional background PBMC RNA at an input of 1 μg (S1 Fig). This observation is supported by previous studies showing that the presence of background RNA is not always inhibitory and can sometimes increase efficiency during the reverse transcription step [94]. The lack of inhibition from cellular RNA suggests that our HIV-2 assays are well suited for testing cellular samples from HIV-2 infected individuals.

At the same time, it should be remembered that a few of our primers/probes do not match the consensus sequence of HIV-2 group B. Although we tried to design primers and probes that match the consensus of both group A and B, in some cases this was not possible without having too many degenerate bases, which can increase nonspecific binding. In these situations, we chose to match the consensus of group A. However, most of the primers and probes are very well conserved for both group A and B. For example, the probes for the TAR, Long LTR, PolyA, and Read-through regions are all highly conserved for both group A and group B. For individual primers where there are mismatches with group B, these are often limited to one or a few nucleotides in the 5’ portion of the primer, where such changes may not have a significant effect on assay performance. Moreover, ddPCR is less susceptible than qPCR to the effects of sequence mismatches. Therefore, we suspect that most of our assays would actually work quite well for both group A and group B. If there are sequence mismatches that substantially inhibit a given assay, this inhibition may be apparent from the raw ddPCR plots (another advantage of ddPCR), and the individual primer or probe could always be corrected to match group B.

Another limitation of this study is that we were unable to apply these assays to clinical samples from HIV-2 infected individuals. However, based on our data using RNA and DNA standards and the use of consensus sequences to design the assays, these RT-ddPCR assays should be ideally suited for investigating the mechanisms of HIV-2 latency in clinical samples. It is also likely that the designed primer/probe sets will perform well in qRT-PCR assays, although we did not evaluate this question directly.

Assays to quantify and study HIV-2 transcription are needed to bridge the gaps in our understanding of HIV latency, particularly in the context of HIV-2. We previously reported the development of a novel HIV-2 plasma RNA viral load assay, validated for clinical use with the Abbott m2000 platform [91, 92]. Other methods to measure HIV-2 include primer sets directed to gag [99–101] or the LTR-gag junction [102, 103], RT-qPCR methods that target HIV-2 unspliced and multiply-spliced mRNA [104], and ddPCR-based quantification of episomal HIV 2-LTR circles as a potential marker for residual viral replication [105].

Given that even during untreated infection, the majority of HIV-2-infected individuals exhibit very low viral loads [8, 106] and a significant proportion of these behave as ‘elite-controllers’ [107], it has been suggested that HIV-2 could serve as a model for a functional HIV cure [108]. Therefore, quantitative assays that can assess transcriptional features of HIV-2 in infected individuals are critical to advance those efforts.

The HIV-2 “transcription profiling” assays described here could be used to investigate how blocks to HIV-2 transcription vary across different tissue sites [109] and to measure the effects of antiretroviral treatment interruption or therapies designed to disrupt latency (such as latency reversing agents) in HIV-2 infected individuals. This innovative method will help to provide critical new insights into HIV-2 transcription and latency, which could advance efforts towards new therapies aimed at "shock and kill,‴ "block and lock," and other approaches designed to disrupt latent HIV infection or achieve viral eradication or functional cure.

## Supporting information

S1 Fig HIV-2 assay sensitivity in presence of background RNA. The IVT HIV-2 RNA standard was added to a common RT reaction with or without background RNA to achieve a final concentration of 1,000 copies/5μL (the input into each ddPCR well, or “expected copies”). cDNA synthesized in this common RT reaction was subsequently divided equally between ddPCR wells for all HIV-2 targets except gag (not present in IVT standard) to assess the efficiency of each primer/probe set.
(TIF)

S1 Table PCR conditions for droplet digital PCR. (DOCX)

S2 Table False positive ddPCR droplets from no template controls. (DOCX)

S3 Table False positive ddPCR wells from no template controls. (DOCX)

S4 Table Assay reproducibility parameters. (DOCX)
