# Supplementary figures and images for: Novel assays to investigate the mechanisms of latent infection with HIV-2
Source: PLoS One. 2022 Apr 27;17(4):e0267402. doi: 10.1371/journal.pone.0267402 (PMC9045618; doi:10.1371/journal.pone.0267402)

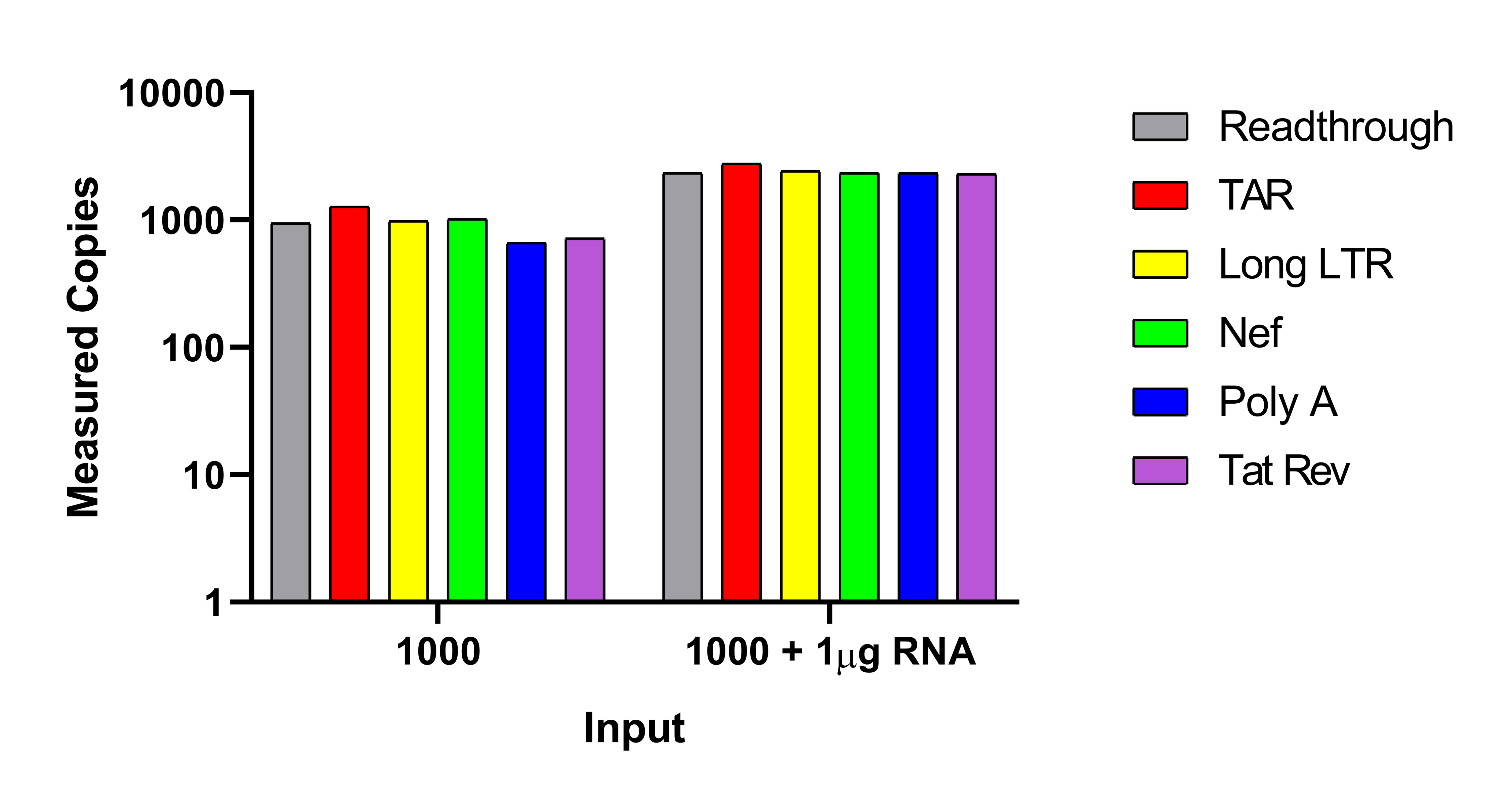

Supplement: S1 Fig — The IVT HIV-2 RNA standard was added to a common RT reaction with or without background RNA to achieve a final concentration of 1,000 copies/5μL (the input into each ddPCR well, or “expected copies”). cDNA synthesized in this common RT reaction was subsequently divided equally between ddPCR wells for all HIV-2 targets except gag (not present in IVT standard) to assess the efficiency of each primer/probe set. (TIF) [file pone.0267402.s001.tif]
