# Supplementary material for: Novel assays to investigate the mechanisms of latent infection with HIV-2
Source: PLoS One. 2022 Apr 27;17(4):e0267402. doi: 10.1371/journal.pone.0267402 (PMC9045618; doi:10.1371/journal.pone.0267402)
Supplement: S1 Table — (DOCX) [file pone.0267402.s002.docx]

**Table S1. PCR conditions for droplet digital PCR**

|  | **Temperature** | **Time** | **Number of Cycles** |
| --- | --- | --- | --- |
| **Enzyme activation** | 95°C | 10 min | 1 |
| **Denaturation** | 95°C | 30 sec | 45 |
| **Annealing/extension** | 59°C | 1 min |  |
| **Enzyme deactivation** | 98°C | 10 min | 1 |
| **Hold** | 4-10°C | infinite | 1 |
