# Supplementary material for: Novel assays to investigate the mechanisms of latent infection with HIV-2
Source: PLoS One. 2022 Apr 27;17(4):e0267402. doi: 10.1371/journal.pone.0267402 (PMC9045618; doi:10.1371/journal.pone.0267402)
Supplement: S2 Table — (DOCX) [file pone.0267402.s003.docx]

**Table S2. False positive ddPCR droplets from no template controls**

| **Assays^1^** | | | | | | | |  |
| --- | --- | --- | --- | --- | --- | --- | --- | --- |
| **Control** | **Readthrough** | **Long LTR** | **TAR** | **Gag** | **Nef** | **Tat-Rev** | **PolyA** | |
| pNL4.3 HIV-1 | 0/31,973 | 1/32,593 | 0/23,177 | 0/29,931 | 0/34,443 | 1/35,342 | 0/32,687 | |
| H_2_O and PBMC RNA | 0/234,101 | 20/280,593 | 1/181,596 | 0/90,053 | 0/168,304 | 0/193,899 | 0/192,120 | |

^1^For each assay, the table shows the total number of false positive droplets (numerator) and total number of accepted droplets (denominator).
