# Supplementary material for: Novel assays to investigate the mechanisms of latent infection with HIV-2
Source: PLoS One. 2022 Apr 27;17(4):e0267402. doi: 10.1371/journal.pone.0267402 (PMC9045618; doi:10.1371/journal.pone.0267402)
Supplement: S3 Table — (DOCX) [file pone.0267402.s004.docx]

**Table S3. False positive ddPCR wells from no template controls**

| **Assays^1^** | | | | | | | |
| --- | --- | --- | --- | --- | --- | --- | --- |
| **Control** | **Readthrough** | **Long LTR** | **TAR** | **Gag** | **Nef** | **Tat-Rev** | **PolyA** |
| pNL4.3 HIV-1 | 0/2 | 1/2 | 0/2 | 0/2 | 0/2 | 1/2 | 0/2 |
| H_2_O and PBMC DNA/RNA | 0/14 | 2/14 | 1/15 | 0/6 | 0/11 | 0/12 | 0/12 |

**^1^**For each assay, the table shows the fraction of wells with at least one positive droplet.
