# Supplementary material for: Novel assays to investigate the mechanisms of latent infection with HIV-2
Source: PLoS One. 2022 Apr 27;17(4):e0267402. doi: 10.1371/journal.pone.0267402 (PMC9045618; doi:10.1371/journal.pone.0267402)
Supplement: S4 Table — (DOCX) [file pone.0267402.s005.docx]

**Table S4. Assay reproducibility parameters**

| **Input** | **~10^2^ copies HIV DNA** | | | | **~10^2^ copies HIV RNA** | | | |  |  |  |
| --- | --- | --- | --- | --- | --- | --- | --- | --- | --- | --- | --- |
|  | Inter-experiment | | | Intra-experiment | Inter-experiment | | | Intra-experiment |  |  |  |
| **Assay** | **Mean of Means^1^** | **Std Dev^2^ of Means** | **% CV^3^ of Means** | **% CV^4^** | **Mean of Means^1^** | **Std Dev of Means^2^** | **% CV of Means^3^** | **% CV^4^** |  |  |  |
| Readthrough | 0.84 | 0.04 | 4.62 | 1.25 | 0.98 | 0.37 | 37.94 | 13.89 |  |  |  |
| TAR | 1.29 | 0.37 | 28.68 | 10.02 | 1.51 | 0.61 | 40.51 | 12.50 |  |  |  |
| LongLTR | 0.82 | 0.08 | 9.75 | 13.65 | 1.05 | 0.43 | 41.00 | 15.88 |  |  |  |
| Nef | 0.76 | 0.14 | 18.17 | 11.67 | 0.80 | 0.20 | 24.62 | 1.59 |  |  |  |
| PolyA | 0.93 | 0.12 | 12.79 | 14.63 | 0.49 | 0.20 | 41.37 | 20.20 |  |  |  |
| Tat-Rev | 0.82 | 0.02 | 1.94 | 22.39 | 0.63 | 0.19 | 30.87 | 9.30 |  |  |  |
|  |  |  |  |  |  |  | | | |  |  |

^1^ For each experiment, measured values were expressed as a fraction of the expected value (measured/expected) and the mean was calculated for all replicates at the 100 copy input. Column shows the mean of the means from all experiments (n=2-4 for DNA, n=4 for RNA).

^2^ Std Dev=standard deviation. Column shows the standard deviation of the means from all experiments

^3^ %CV=coefficient of variability. Column shows the coefficient of variability between experiments, as measured by the Std Dev of Means/Mean of Means*100.

^4^ For each experiment, the %CV was calculated as the standard deviation of all replicates at the 100 copy input divided by the mean of all replicates*100. Column shows the average of the %CV from all experiments.

| **Input** | **~10^3^ copies HIV DNA** | | | | **~10^3^ copies HIV RNA** | | | |
| --- | --- | --- | --- | --- | --- | --- | --- | --- |
|  | Inter-experiment | | | Intra-experiment | Inter-experiment | | | Intra-experiment |
| **Assay** | **Mean of Means^5^** | **Std Dev^6^ of Means** | **% CV^7^ of Means** | **% CV^8^** | **Mean of Means^5^** | **Std Dev of Means^6^** | **% CV of Means^7^** | **% CV^8^** |
| Readthrough | 0.82 | ND^9^ | ND | 1.03 | 0.82 | 0.17 | 21.26 | 7.73 |
| TAR | 1.26 | 0.26 | 20.89 | 5.47 | 1.45 | 0.19 | 13.16 | 9.71 |
| LongLTR | 0.77 | 0.08 | 10.92 | 5.79 | 0.93 | 0.18 | 19.27 | 15.50 |
| Nef | 0.93 | 0.06 | 6.87 | 3.48 | 1.021 | ND | ND | 1.52 |
| PolyA | 1.01 | ND | ND | 2.00 | 0.52 | 0.21 | 40.36 | 2.31 |
| Tat-Rev | 0.75 | ND | ND | 6.82 | 0.66 | 0.08 | 12.24 | 4.92 |

^5^ For each experiment, measured values were expressed as a fraction of the expected value (measured/expected) and the mean was calculated for all replicates at the 1000 copy input. Column shows the mean of the means from all experiments (n=3 for DNA, n=2 for RNA unless indicated by “ND”).

^6^ Std Dev=standard deviation. Column shows the standard deviation of the means from all experiments.

^7^ %CV=coefficient of variability. Column shows the coefficient of variability between experiments, as measured by the Std Dev of Means/Mean of Means*100.

^8^ For each experiment, the %CV was calculated as the standard deviation of all replicates at the 1000 copy input divided by the mean of all replicates*100. Column shows the average of the %CV from all experiments.

^9^ ND: Not determined. For some assays, the 1000 copy input was only tested in one experiment.
